# Role for Gag-CA Interdomain Linker in Primate Lentiviral Replication

**DOI:** 10.3389/fmicb.2019.01831

**Published:** 2019-08-07

**Authors:** Naoya Doi, Takaaki Koma, Akio Adachi, Masako Nomaguchi

**Affiliations:** ^1^Department of Microbiology, Graduate School of Medical Science, Tokushima University, Tokushima, Japan; ^2^Department of Microbiology, Kansai Medical University, Osaka, Japan

**Keywords:** HIV/SIV, Gag-CA, interdomain linker, Gag assembly, virus production, early infectivity, virus replication

## Abstract

Gag proteins underlie retroviral replication by fulfilling numerous functional roles at various stages during viral life cycle. Out of the four mature proteins, Gag-capsid (CA) is a major component of viral particles, and has been most well studied biogenetically, biochemically and structurally. Gag-CA is composed of two structured domains, and also of a short stretch of disordered and flexible interdomain linker. While the two domains, namely, N-terminal and C-terminal domains (NTD and CTD), have been the central target for Gag research, the linker region connecting the two has been poorly studied. We recently have performed systemic mutational analyses on the Gag-CA linker region of HIV-1 by various experimental and *in silico* systems. In total, we have demonstrated that the linker region acts as a *cis*-modulator to optimize the Gag-related viral replication process. We also have noted, during the course of conducting the research project, that HIV-1 and SIVmac, belonging to distinct primate lentiviral lineages, share a similarly biologically active linker region with each other. In this brief article, we summarize and report the results obtained by mutational studies that are relevant to the functional significance of the interdomain linker of HIV/SIV Gag-CA. Based on this investigation, we discuss about the future directions of the research in this line.

## Introduction

Gag is a main viral protein initially synthesized as a precursor in cells infected with retroviruses (Freed and Martin, 2013; Goff, 2013). Gag precursors then multimerize at the plasma membrane (PM) to form immature virus particles with other viral and cellular components, followed by their extracellular release and final maturation via processing by viral protease (Freed and Martin, 2013; Goff, 2013). Although much about those events remains to be mechanistically elucidated, correct assembly of precursor Gag proteins in cells and the subsequent proper maturation at the final stage are certainly prerequisites for the generation of retroviral infectious virions and for the next round of successful productive infection in target cells. HIV-1 Gag-CA mutants that form aberrant capsid core or with defects in virus production simultaneously exhibit a severe reduction in the early viral infectivity (von Schwedler et al., 2003; Chang et al., 2007; Jiang et al., 2011; Robinson et al., 2014; Tanaka et al., 2016; Koma et al., 2019). Gag-CA, mainly consisting of structured NTD and CTD, plays a major role in the above processes, and thus is essential for both early and late viral replication phases. It is well established from extensive mutational and structural studies that HIV-1 Gag-CA carries out its pivotal functions in a sophisticatedly regulated way (Sundquist and Kräusslich, 2012; Bell and Lever, 2013; Lingappa et al., 2014; Campbell and Hope, 2015; Freed, 2015; Mattei et al., 2016).

Assembly of immature precursor Gag proteins in cells is mainly driven by their Gag-CA domain. Following synthesis in the cytoplasm, the precursors gradually multimerize in due course. Recent structural studies on the immature HIV-1 Gag-CA have demonstrated that intra-hexameric NTD-CTD, inter-hexameric NTD-NTD, inter-hexameric CTD-CTD, and intra-hexameric CTD-CTD contacts can be formed during the assembly (Bharat et al., 2012, 2014; Schur et al., 2015, 2016). Of note, mutations that cause the defect in virus production resided within or adjacent to the Gag-CA contact sites described above. Thus, it can be concluded that various amino acid residues and/or regions in both CA-NTD and CA-CTD are critical for the Gag assembly and subsequent virus particle production.

While much is known about the role and activity of CA-NTD and CA-CTD as described above, research reports on the interdomain linker region itself was limited (Arvidson et al., 2003; Jiang et al., 2011). Some studies on a serine residue in the linker region (amino acid number 149 for HIV-1 Gag-CA) were conducted (Cartier et al., 1999; Wacharapornin et al., 2007; Brun et al., 2008; Takeuchi et al., 2017), mainly because it is a major phosphorylation site in HIV-1 Gag-CA. Totally, it was quite unclear how important or critical the interdomain linker region of HIV-1 Gag-CA is for Gag assembly, virus production, and viral early infectivity. Thus, much remains to be clarified about the role for the CA-linker region in virus replication cycle. In this report, we sort out important functional and structural information on the interdomain linker region of HIV/SIV Gag-CA published so far, and enrich the discussion by summarizing our new report just published (Koma et al., 2019) and by adding new relevant data on the corresponding CA-linker mutants of a standard SIVmac clone.

## Comparison of Amino Acid Sequences in the Linker Region of SIV_*Mac*_ and HIV-1 Gag-CA Proteins

**FIGURE 1:**
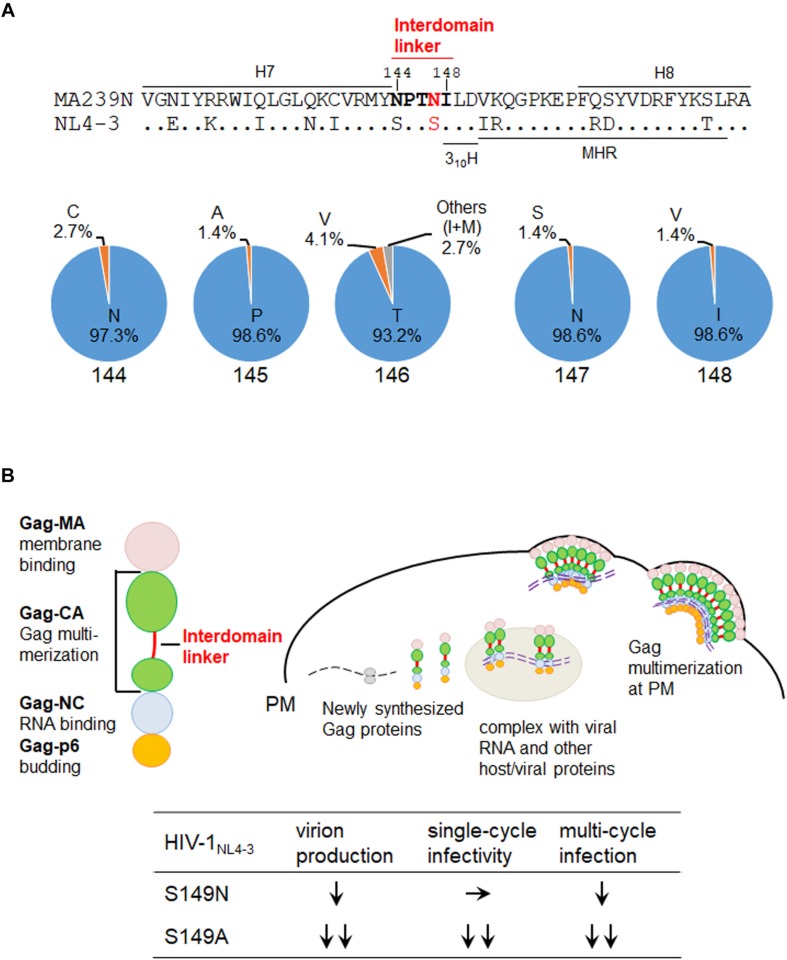
Interdomain linker region of SIV/HIV Gag-CA proteins. **(A)** Amino acid residues in the Gag-CA linker region. Upper: the amino acid sequence alignment of the Gag-CA region of SIVmac clone MA239N (Shibata et al., 1991; Doi et al., 2010, 2011) and HIV-1 clone NL4-3 (Adachi et al., 1986) that encompasses the helix 7 (H7), interdomain linker, helix 3_10_H (Gres et al., 2015), major homology region (MHR), and H8. Amino acids in the SIVmac Gag-CA linker region are highlighted by bold letters. In addition, the amino acid site into which mutations were introduced in this study is indicated by red letters. Amino acid numbers are those for SIVmac239 (GenBank: M33262). Lower: the frequencies of amino acids at numbers 144 to 148 (the linker region of HIV-2/SIV) for various HIV-2/SIVs. These Data are based on the sequences of 74 clones listed in the HIV-2/SIV Proteins of the HIV Sequence Compendium 2018 (https://www.hiv.lanl.gov/content/sequence/HIV/COMPENDIUM/2018/sequence2018.pdf). **(B)** Intracellular assembly process of HIV-1 Gag precursor protein and replication phenotypes of relevant HIV-1_NL4–3_ linker mutants. Major function for each of the four Gag domain is also shown on the left. HIV-1 Gag assembly process in cells is schematically shown. Previously reported phenotypes of the HIV-1 interdomain linker mutants designated S149N and S149A (Koma et al., 2019) are summarized at the bottom. S149A is more attenuated than S149N as shown. S149A, ∼12% and ∼6% of wild-type NL4-3 for the virion production and single-cycle infectivity, respectively; S149N, ∼46% and ∼99% of NL4-3 for the virion production and single-cycle infectivity, respectively (Koma et al., 2019). MA, matrix protein; CA, capsid protein; NC, nucleocapsid protein; p6, p6 protein; PM, plasma membrane.

We recently have analyzed the interdomain linker region of HIV-1 Gag-CA in detail (Koma et al., 2019). The amino acid sequence of the region is SPTSI (Figure 1A), and as reported (Koma et al., 2019), is remarkably well conserved among HIV-1 isolates (98.9 to 99.9% for each residue in subtype B viruses and 97.5 to 99.9% in group M viruses) except for the T residue in the middle (amino acid numbers 146 and 148 for SIVmac MA239N and HIV-1 NL4-3 numbering systems, respectively) (64.1% for subtype B viruses and 31.6% for group M viruses). The fact that the T residue is significantly more conserved for subtype B viruses than for group M viruses may imply that the “SPTSI” sequence emerged following inter-species transmission of SIVcpz to humans. In fact, only CPVGI, CPVSI, SPASI, and SPVSI are identified in the SIVcpz sequences (sequences of 13 clones listed in the HIV-1/SIVcpz Proteins of the HIV Sequence Compendium 2009^1^ and 2018^2^). The corresponding sequence of SIVmac Gag-CA (NPTNI in Figure 1A) was also conserved well (93.2 to 98.6% for each residue) (sequences of 74 clones listed in the HIV-2/SIV Proteins of the HIV Sequence Compendium 2018; see footnote 2). Although not so drastic when compared with the HIV-1 linker, we noted that the frequency of the T residue at the 146th position is relatively lower (93.2%) than those of the amino acids at the other positions (97.3 to 98.6%). Interestingly, most of the 3rd amino acid in the linker region is T or V residue: 89.6% for HIV-1 subtype B and 94.9% for HIV-1 group M; 97.3% for HIV-2/SIV. Another point worth mentioning about the HIV-1 Gag-linker here is that different effects of amino acid types substituted at the 149th position (147th for SIVmac Gag) on HIV-1 replication properties (Koma et al., 2019). Mutant S149N could grow albeit poorly in target lymphocytic cells, but mutants S149A, S149D, and S149K did not grow at all. Consistently, S149N exhibited normal early single-cycle infectivity in indicator cells but was partially defective for virus production (late replication phase). S149A, S149D, and S149K were severely defective or negative for the early infectivity and viral particle production. Thus, the S to N substitution at the 149th position (147th for SIVmac239 in Figure 1A) only gave a mild mutational effect, and S149N showed a less attenuated phenotype than S149A, S149D, and S149K. The results on viral replication properties obtained for mutants S149N and S149A (Koma et al., 2019) are summarized at the bottom of Figure 1B. While S149A was very attenuated (∼12% and ∼6% relative to wild-type NL4-3 for the virion production and single-cycle infectivity, respectively), S149N considerably or comparably retained the viral activities (∼46% virion production and ∼99% single-cycle infectivity of NL4-3) (Koma et al., 2019).

## Structural Regulation of HIV Gag-CA for Viral Replication by Its Interdomain Linker Region

Molecular events for HIV-1 Gag-CA assembly are schematically summarized in Figure 1B. In our recent work, we have extensively characterized the interdomain linker mutants of HIV-1 Gag-CA by various virological, biochemical, and molecular biological methods (Koma et al., 2019) to see if and how the mutations affect the assembly process using the virus production level as a final outcome. Out of nine linker mutants analyzed, eight were found to be defective for virus particle production, to various degrees, from lymphocytic producer cells relative to wild-type NL4-3. Some mutants were similarly deficient with controls that are known not to produce virus particles (von Schwedler et al., 2003; Robinson et al., 2014; Tanaka et al., 2016). Together with the observation that Gag mutations affecting the virus production have little effects on the expression in cells of Gag itself, it was quite clear that the linker region acts on or modulate the Gag assembly process. It was also evident from the analyses and previous data that the linker mutants with severely reduced early infectivity are unable to produce morphologically normal virus particles (Jiang et al., 2011; Koma et al., 2019). We next examined the linker mutants for the Gag-targeting to the PM by biochemical (sucrose density gradient centrifugation) and confocal microscopic analyses. As indicated in Figure 1B, Gag-NC and Gag-MA mediates the Gag-viral RNA binding and Gag-PM binding, respectively (Sundquist and Kräusslich, 2012; Freed, 2015). Our experimental results on this issue showed that the decrease in virus production for the linker mutants is primarily due to the impaired Gag-Gag interactions at PM, although they exhibit inefficient PM binding and localization to some extent. Consistent with this conclusion, the live-cell protein interaction analysis, NanoBRET analysis, demonstrated that Gag proteins of virus production-defective mutants (S149A, S149D, and I150A) are impaired with interacting activity as negative control mutants (major homology region (MHR) mutants K158A and Y164A), but not of the mutants with normal or moderately defective phenotype (S146A, S146N, and S149N) with respect to virus production. Whether the mutants with defects in the virion production show dominant-negative effects on the normal replication process of wild-type virus remains to be experimentally analyzed. This information may be of value for potential therapeutic application in future. In summary, our results strongly suggest that the interdomain linker of HIV-1 Gag-CA modulate the Gag-Gag interaction/Gag assembly at PM. However, it should be noted that mutational effects of the region are relatively mild compared with those of NTD and CTD (Koma et al., 2019).

Since the hydrophobic interplay is known to be a major factor contributing to the protein-oligomerization (Janin et al., 1988; Jones and Thornton, 1996; Chiti et al., 2003; Jacak et al., 2012), we examined 3D distributions of hydrophobic patches on HIV-1 Gag-CA (Koma et al., 2019). We found that hydrophobic patches along or besides CA-CA interfaces are present on immature Gag-CA, and that their distribution is different between immature and mature Gag-CA proteins. Taken together, it was suggested that the hydrophobic patches may play important roles in both early and late viral replication phases. We finally analyzed molecular bases that underlie the activity of interdomain linker region by *in silico* system (Koma et al., 2019). We were interested in predicting how the region modulates the Gag assembly process. Extensive molecular dynamics (MD) simulations of wild-type (NL4-3) and mutant (linker mutants S146A, S149A, I150A, and MHR mutant Y164A) Gag-CA proteins have indicated that the linker region and MHR can remotely affect structural fluctuations of immature and mature Gag-CA interaction surfaces (allosteric regulation of HIV-1 Gag-CA structure).

## Replication Phenotypes of SIV Gag-CA Linker Mutants

**FIGURE 2:**
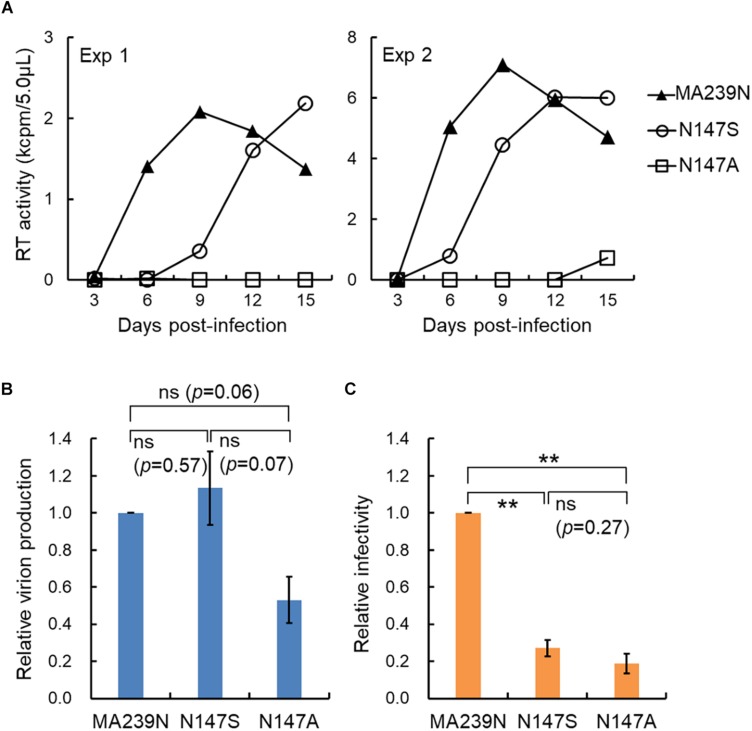
Replication ability of SIVmac mutants with site-specific mutations in the Gag-CA linker region. **(A)** Growth kinetics of the linker mutants (N147S and N147A) in rhesus macaque M1.3S cells. Input viruses including a wild-type MA239N clone, prepared from transfected 293T cells (Nomaguchi et al., 2014, 2016), were inoculated into M1.3S cells (Doi et al., 2011), and infected cells were monitored for virus replication at intervals by virion-associated reverse transcriptase (RT) (Willey et al., 1988; Nomaguchi et al., 2013) activity in the culture supernatants. Amounts of input viruses were normalized by RT. Infection condition: 1 × 10^4^ RT units/2 × 10^5^ cells. Four independent infection experiments were performed, and representative results were presented (experiments 1 and 2). **(B)** Virus production level in transfected 293T cells. Proviral DNA clones indicated (5 μg) were transfected into 293T cells as previously described (Nomaguchi et al., 2016), and on day 2 post-transfection, culture supernatants were collected for RT assays. Virus production level relative to that for wild-type clone (SIVmav239N) is shown. *n* = 3. Statistical significance was determined by Welch’s *t*-test. ns, statistically not significant. *P*-values for each pair are indicated. **(C)** Early infectivity in TZM-bl cells of the linker mutants. To determine viral infectivity, virus samples (2 × 10^3^ RT units prepared from transfected 293T cells) were inoculated into TZM-bl cells (4 × 10^3^ cells) (Platt et al., 1998, 2009), and cell lysates were prepared for luciferase assays (Promega) on day 2 post-infection as previously described (Nomaguchi et al., 2016; Koma et al., 2019). Infectivity relative to that of wild-type clone (SIVmac239N) is shown. *n* = 3. Statistical significance was determined by Welch’s *t*-test. ns, statistically not significant. *P*-value for N147S vs. N147A is indicated. ^∗∗^, *p* < 0.01.

We recently have determined various phenotypes of the interdomain linker mutants of HIV-1 Gag-CA, and proposed the functional role of the linker region for viral replication (Koma et al., 2019). The phenotypes of the linker mutants can be summarized as follows: (i) progeny virus production is markedly or moderately reduced; (ii) viral early infectivity is considerably or drastically impeded; (iii) progression of Gag multimerization at PM is impaired while certain amounts of membrane-bound Gag accumulates; (iv) mutational effects are generally mild; (v) mutational effects are site- and amino acid-dependent in the linker region. For confirmative and comparative purpose, it is of great value to determine the replication phenotype of interdomain linker mutants of SIVmac Gag-CA. The results and discussions described above point out important issues from the general to the particular: functional details of the linker region for Gag assembly and individual points related to viral mutational assays. We focused here the amino acid residue N at position 147 (S at position 149 for HIV-1) (Figure 1A). We constructed SIVmac proviral mutants N147S and N147A, and compared their virological properties with those of corresponding HIV-1 mutants S149N and S149A. As shown in Figure 2A, the two SIVmac Gag-CA mutants grew very differently in macaque M1.3S cells, quite similarly as reported for HIV-1 S149N and S149A mutants in human H9 cells (Koma et al., 2019). Of note, N147S (SIVmac) and S149N (HIV-1) were infectious, whereas N147A (SIVmac) and S149A (HIV-1) were faintly infectious or non-infectious. It is possible that the progeny virus populations from N147S-infected cells contained revertants and/or mutants with additional mutations. Although it is unlikely that such infectious variants emerged and constituted a major population in a short period of time as can be seen in Figure 2A, the genomes of progeny viruses need to be analyzed to confirm this. The abilities of the two SIVmac mutants to produce progeny virus particles upon transfection were also different (Figure 2B). Although statistically not significant, there was an obvious difference between the results for the two mutants. Because M1.3S cells are too fragile for transfection analysis, 293T cells were used for the experiments instead. The 293T cells are very sensitive to transfection assays, and can produce a considerable amount of virus particles even for the mutants defective in the virion production. Thus, we may have overestimated the relatively poor ability of N147A to produce virus particles. In fact, while viral early infectivity was similarly impaired for the two mutants (Figure 2C), N147S grew much more efficiently than N147A (Figure 2A). This result is certainly attributable to the difference in virus-producing ability of the two mutant viruses (Figure 2B), being essentially similar as observed for the corresponding two HIV-1 mutant viruses (Figure 1B). Totally, viral replication properties of HIV-1 and SIVmac mutants with mutations in a specific and corresponding site in the interdomain linker region were in parallel except for the infectivity (compare the mutational effects on viral infectivity of S149N and N147S in Figures 1B and 2C, respectively). As for this difference, it is not unreasonable to assume that mature CA-CA interaction surfaces of HIV-1 and SIVmac were differently influenced by each linker mutation in each backbone Gag-CA sequence. In essence, our results suggest that the linker region of the two viruses, HIV-1 and SIVmac of different primate lentiviral lineages, has a similarly important role for the Gag assembly process. Thus, the interdomain linker may probably be evolutionarily conserved, and is important region for Gag-related events, and finally for viral replication.

## Concluding Remarks

Major conclusion in this report is that the interdomain region of primate lentivirus Gag-CA plays an important role for viral replication. More decisively and accurately, the Gag-linker region of HIV-1 and probably of SIVmac is a *cis*-acting allosteric modulator of CA interacting surfaces. Experimental and *in silico* evidence supports the notion that the linker region optimizes Gag assembly, progeny virus production, and viral early infectivity by remotely regulating various interactions among CA-NTD and CA-CTD (Koma et al., 2019; this report). Of note, the interdomain linker connecting two structured domains of Hsp70, a totally distinct cellular protein from viral Gag-CA, has been recently reported to act as a dynamic switch that allosterically regulates the interaction between the two domains (English et al., 2017).

There are a number of scientific issues to be addressed. (i) The disordered linker region is considered to remotely regulate the overall structural dynamics and molecular interactions of Gag-CA. Then, how the linker region can allosterically modulate the dynamics of Gag-CA? It is possible that networks of hydrophobic and hydrophilic interactions on CA surface influence the mobility of amino acids away from the linker region. (ii) How does the Gag-precursor multimerization start? Factor(s) involved in the initiation process is not yet fully uncovered (Sundquist and Kräusslich, 2012; Lingappa et al., 2014; Freed, 2015). This issue should be definitively elucidated. (iii) The 3rd amino acid in the linker region is relatively variable (mostly T or V residue). What is the molecular basis for the variability? How advantageous is it for viruses? The variability may be necessary to optimize virus growth ability in the context of the backbone Gag-CA sequence. (iv) The linker region is conserved among HIV/SIV with respect to sequence and activity. Then, how about the other retroviruses? Do viruses other than those of the retrovirus family have this kind of allosteric regulation system? It is also intriguing to test functional exchangeability of various “disordered linker regions.” This would help to understand mechanistic and biological basis for the linker domain in various viral and non-viral proteins.

It is absolutely important, in terms of basic virology and also of developing new anti-HIV drugs, to fully understand the molecular mechanism for the Gag-CA linker’s action and determine viral and cellular factors involved in the relevant process. Studies in this line are in progress in our laboratory.

## Data Availability

The datasets generated for this study are available on request to the corresponding author.

## Author Contributions

MN designed the research project. ND, TK, and MN performed the experiments. All authors discussed the results and approved the submission. AA and MN wrote the manuscript.

## Conflict of Interest Statement

The authors declare that the research was conducted in the absence of any commercial or financial relationships that could be construed as a potential conflict of interest.

## Footnotes

1 https://www.hiv.lanl.gov/content/sequence/HIV/COMPENDIUM/2009/hiv1prot.pdf

2 https://www.hiv.lanl.gov/content/sequence/HIV/COMPENDIUM/2018/sequence2018.pdf
